# Data Quality of Longitudinally Collected Patient-Reported Outcomes After Thoracic Surgery: Comparison of Paper- and Web-Based Assessments

**DOI:** 10.2196/28915

**Published:** 2021-11-09

**Authors:** Hongfan Yu, Qingsong Yu, Yuxian Nie, Wei Xu, Yang Pu, Wei Dai, Xing Wei, Qiuling Shi

**Affiliations:** 1 School of Public Health and Management Chongqing Medical University Chonqqing China; 2 State Key Laboratory of Ultrasound in Medicine and Engineering, College of Biomedical Engineering Chongqing Medical University Chongqing China; 3 Department of Thoracic Surgery Sichuan Cancer Hospital & Institute, Sichuan Cancer Center School of Medicine, University of Electronic Science and Technology of China Chengdu, Sichuan China

**Keywords:** patient-reported outcome (PRO), data quality, MDASI-LC, postoperative care, symptoms

## Abstract

**Background:**

High-frequency patient-reported outcome (PRO) assessments are used to measure patients' symptoms after surgery for surgical research; however, the quality of those longitudinal PRO data has seldom been discussed.

**Objective:**

The aim of this study was to determine data quality-influencing factors and to profile error trajectories of data longitudinally collected via paper-and-pencil (P&P) or web-based assessment (electronic PRO [ePRO]) after thoracic surgery.

**Methods:**

We extracted longitudinal PRO data with 678 patients scheduled for lung surgery from an observational study (n=512) and a randomized clinical trial (n=166) on the evaluation of different perioperative care strategies. PROs were assessed by the MD Anderson Symptom Inventory Lung Cancer Module and single-item Quality of Life Scale before surgery and then daily after surgery until discharge or up to 14 days of hospitalization. Patient compliance and data error were identified and compared between P&P and ePRO. Generalized estimating equations model and 2-piecewise model were used to describe trajectories of error incidence over time and to identify the risk factors.

**Results:**

Among 678 patients, 629 with at least 2 PRO assessments, 440 completed 3347 P&P assessments and 189 completed 1291 ePRO assessments. In total, 49.4% of patients had at least one error, including (1) missing items (64.69%, 1070/1654), (2) modifications without signatures (27.99%, 463/1654), (3) selection of multiple options (3.02%, 50/1654), (4) missing patient signatures (2.54%, 42/1654), (5) missing researcher signatures (1.45%, 24/1654), and (6) missing completion dates (0.30%, 5/1654). Patients who completed ePRO had fewer errors than those who completed P&P assessments (ePRO: 30.2% [57/189] vs. P&P: 57.7% [254/440]; *P*<.001). Compared with ePRO patients, those using P&P were older, less educated, and sicker. Common risk factors of having errors were a lower education level (P&P: odds ratio [OR] 1.39, 95% CI 1.20-1.62; *P*<.001; ePRO: OR 1.82, 95% CI 1.22-2.72; *P*=.003), treated in a provincial hospital (P&P: OR 3.34, 95% CI 2.10-5.33; *P*<.001; ePRO: OR 4.73, 95% CI 2.18-10.25; *P*<.001), and with severe disease (P&P: OR 1.63, 95% CI 1.33-1.99; *P*<.001; ePRO: OR 2.70, 95% CI 1.53-4.75; *P*<.001). Errors peaked on postoperative day (POD) 1 for P&P, and on POD 2 for ePRO.

**Conclusions:**

It is possible to improve data quality of longitudinally collected PRO through ePRO, compared with P&P. However, ePRO-related sampling bias needs to be considered when designing clinical research using longitudinal PROs as major outcomes.

## Introduction

### Scientific Background

Patient-reported outcomes (PROs) are commonly assessed as primary or secondary outcomes in clinical trials or observational studies to evaluate the effect of medical interventions from the viewpoint of patients without interpretation by professionals [1-3]. PROs can help clinicians monitor adverse events [4], relieve symptom burdens [5], guide clinical care [4,6], and improve patient outcomes [6], such as quality of life (QOL) and survival. However, PROs involve multiple self-evaluations over time, and symptoms change frequently over the course of treatment in clinical studies [7]. Especially in surgical research and practice, daily assessments have been used to precisely describe the trajectory of symptom relief and functional recovery because the daily changes in symptoms in surgical patients have been found to be statistically significant [8-10]. However, whether the high frequency of assessment affects data quality has seldom been discussed in studies using longitudinal PROs as major outcomes.

What does “data quality” actually mean? Wang and Strong [11] and Kahn et al [12] proposed that data should be of sufficient quality to be of use to data consumers pursuing specific goals. For longitudinal data repositories, Weiskopf et al [13] characterized “data quality” as completeness. Charnock [14] conducted a systematic review in 2019 and reported that all papers referred to the importance of accuracy and completeness when evaluating data quality. Currently, data quality evaluations in longitudinal studies have focused on missing assessments [15-17]. However, other issues, such as item nonresponse and sample bias, have emerged over time [18,19], and these issues may impact data availability and consistent interpretations. Recent studies reported that repeated source data verification could improve accuracy and completeness by 40% [19], and better data quality could improve epidemiological inferences [20]. Additionally, partly due to the lack of an international definition of “error” [21], very few descriptions of the determinants of poor data quality have been provided in clinical studies [22]. Thus, there is an urgent need to characterize types of errors and the factors that affect longitudinal data quality to enable more interpretable results to be obtained from more complete data.

Paper-and-pencil (P&P) or electronic-based assessment of PRO (electronic PRO [ePRO]) are the 2 common modes used in clinical practice [23,24]. Compared with the P&P method, ePRO is more likely to generate complete data [17]; results in fewer data entry errors [25]; is more user friendly [26]; results in a shorter turnaround time [27]; and allows data to be processed, reviewed, and disseminated quickly [28,29]. Currently, interactive ePRO assessments can provide immediate feedback from patients [30] and are a convenient means of monitoring patients and delivering early warnings to clinicians [31,32]. In surgical research, due to the daily changes in symptoms after surgery [8-10], daily ePRO assessments have been used to precisely describe symptom relief and functional recovery. However, the often-mentioned disadvantages of ePRO assessments are sample bias [15,22,33] and a lower response rate [15-17,33]. Thus, generating a profile of the quality of data obtained with P&P and ePRO assessments will guide the appropriate selection of the mode of assessment.

### Objectives

Daily PRO data collected via either P&P or ePRO assessments over the course of recovery from thoracic surgery for malignant or benign lung tumors were used in this analysis, with the following aims: (1) to describe error patterns in PRO data collected via the 2 major PRO measurement modes (ie, P&P and ePRO); (2) to identify factors influencing the incidence of errors; and (3) to generate profiles of the trajectories of errors over the course of a high-frequency data collection schedule.

## Methods

### Data Sources

Data were extracted from 2 prospective studies: 1 observational study [34] and 1 randomized controlled trial (RCT) [35]. The 2 original studies were approved by the Ethics Committee of Sichuan Cancer Hospital (No. SCCHEC-02-2017-042 and No. SCCHEC-02-2018-045).

All patients were assessed with the MD Anderson Symptom Inventory Lung Cancer Module (MDASI-LC) [36] and the single-item QOL scale [37] within 3 days before surgery and then daily after surgery until discharge or for up to 14 days if the patient stayed in the hospital for longer than 14 days after surgery. The MDASI-LC consists of 2 parts. Part I includes not only items regarding 13 core symptoms but also 3 items specific to lung cancer. Part II includes 6 interference items.

All data collection communications with medical staff were conducted face-to-face, and reminders were provided in the hospitals. Patients were asked to consider their symptoms over the previous 24 hours. When a participant completed and submitted a survey, he or she was not able to later modify the answers. On P&P assessments, signatures and data were collected from the patients and researchers for each record. Any time a patient modified a P&P form, the patient was asked to sign below the modified item. Assessment through ePRO only required the patient’s e-signature for each record.

The observational study used P&P, ePRO, phone-to-paper, and mixed assessments, while the RCT used only ePRO assessments. All PRO data were stored in the REDCap [38,39] online management system. EPRO data were automatically imported into REDCap within 24 hours, whereas the P&P forms were manually entered into this platform. Both studies were approved by the ethics committees of all participating hospitals. All participants signed informed consent forms [34,35].

For the P&P assessments, the original paper questionnaires were first checked by the data collectors for amendable errors (eg, missing researcher signatures at the end of completed questionnaires). After both the P&P and ePRO data were entered into REDCap, the database was closed and sent for a data audit by a third team. The classification of errors was performed by 2 independent data management experts (QS and WD) with experience in clinical research data management. Inconsistencies were discussed within the audit team to reach a consensus. Data with errors identified during the audit were then entered into an electronic database in REDCap by 2 independent investigators (HY and QY) and cross-checked. The audit included (1) the withdrawal rate of each study; (2) patient compliance with the scheduled times of the assessments; (3) the completeness and accuracy of PRO forms with regard to individual items; and (4) rate of missing signatures and dates of completion.

### Outcome Defined

Six types of errors were summarized into 2 groups, namely, incompleteness and inaccuracy, and used as indicators of PRO data quality:

- Incompleteness: any missing (1) individual items; (2) patient signatures; (3) researcher signatures; or (4) dates of completion.
- Inaccuracy: any (1) multiple selections for 1 item or (2) missing patient signature on any modified answer.

When any type of error mentioned above was found for any item on the MDASI-LC or QOL scale, it was counted as 1 error, and the corresponding patient was defined as a *patient with error*. A record of an error was defined as any error found for each PRO instrument (MDASI-LC or single-item QOL). A time point with any error in the record was labeled a *time point with an error*. Overall errors refer to all errors of all types in all records.

### Data Analysis and Management

Reporting was performed according to STROBE guidelines [40]. To be included in the analysis, a patient must have provided PRO data at baseline and at least one additional time point during follow-up. We used the mean (SD) or median (range) for continuous variables and frequency (%) for categorical variables to describe the variables. Differences were analyzed using the 2-sample independent *t*-test, 2-sample Wilcoxon test, chi-square test, and Fisher exact test as appropriate. The withdrawal rate refers to the proportion of patients who did not provide a response to the assessment prior to the day of discharge. Patient compliance was calculated as the number of PRO assessments returned divided by the number of PRO assessments that should have been returned. We analyzed at most 8 time points (1 time before surgery and 7 days after surgery) when creating the profiles of the trajectories of the errors over time.

A multivariate generalized estimating equation (GEE) model was constructed to select and estimate the associations between potential risk factors and the incidence of errors for each mode of assessment. The factors included age (≤55 year vs. >55 year), sex (male vs. female), education (median school graduate or below vs. above), employment status (employed vs. other), surgical approach (video-assisted thoracoscopic surgery [VATS] vs. thoracotomy), hospital type (provincial vs. municipal or county level), BMI (≤23.9 kg/m^2^ vs. >23.9 kg/m^2^), smoking status (yes vs. no), Charlson Comorbidity Index (CCI) score (≤1 vs. >1), number of chest tubes (1 vs. 2), disease type (not-lung cancer vs. lung cancer with pathological tumor–node–metastasis [pTNM] stage>I, lung cancer with pTNM stage ≤I vs. lung cancer with pTNM stage>I), and postoperative hospital stay days (>6 vs. ≤6). The effect of risk factors is presented as odds ratios (ORs) with 95% CIs. Using Bonferroni correction [41] for multiple comparisons of risk factor identification, statistical significance level was set at the adjusted cutoff of *P*<.004, adjusted by the number of risk factors (0.05/number of risk factors).

The GEE model was also used to describe the trajectories of the incidences of errors over the 7 time points after surgery between those who used the P&P and ePRO assessments. The incidence of all errors or missing items was the dependent variable and the baseline covariates (the significant variables in the previous GEE model analysis), days after surgery (as a continuous variable), assessment modes, and the interaction between time and assessment mode were the independent variables. The binomial distribution and logit link function were adopted in all models. Co-variance structure types, such as unstructured, autoregressive, independent, exchangeable, and compound symmetric, were compared via quasi-likelihood under the independence model criterion (QIC). The models with QICs closest to 0 were closed as the final models. Two-piecewise random coefficient models were used to analyze trends before and after surgery. Time points with the highest proportion of errors were defined as the change points in the 2-piecewise models. All *P* values were 2 tailed, and statistical significance was set at the conventional cutoff of *P*<.05. All data analyses were performed using the statistical software SAS (version 9.4; SAS Institute).

## Results

### Participants

We extracted data pertaining to patients scheduled for lung surgery from the observational study (n=512) and the RCT (n=166). Thirty-six patients were excluded because they used phone-to-paper or mixed assessments, and 13 patients had only 1 PRO record. Finally, 629 patients responded to either P&P (n=440) or ePRO (n=189) assessments.

Patient characteristics are presented in Table 1. Compared with those using P&P assessments, patients using ePRO assessments were younger (51.5 vs. 55.5; *P*<.001), had higher levels of education (67.2% [127/189] vs. 50.0% [220/440]; *P*<.001), lower CCI scores (75.7% [143/189] vs. 60.7% [267/440]; *P*<.001), earlier stages of disease (compare with lung cancer with pTNM stage >I, 85.2% [161/189] vs. 68.0% [299/440]; *P*<.001), were more likely to have undergone VATS (93.1% [176/189] vs. 81.4% [358/440]; *P*<.001), and had shorter postoperative hospital stay (5 days vs. 6 days; *P*<.001). However, the differences in employment status, hospital type, and BMI were not significant (Table 1).

**Table 1:** Baseline characteristics of participants who filled out the P&P^a^ or ePRO^b^ assessment.^c^

Variables			ePRO (n=189)	P&P (n=440)	*P* value^d^
Age (years), mean (SD)			51.5 (10.8)	55.5 (10.3)	*<.001* ^e^
Postoperative hospital stay (days), median (range)			5 (1-25)	6 (2-41)	*<.001* ^f^
**Gender, n (%)**					*<.001* ^g^
		Male	73 (38.6)	247 (56.1)	
		Female	116 (61.4)	193 (43.9)	
**Education** **, n (%)**					*<.001* ^g^
	Middle school or below		62 (32.8)	220 (50.0)	
	Higher than middle school		127 (67.2)	220 (50.0)	
**Employment status** **, n (%)**					.62^g^
	Employed		85 (45.0)	198 (45.0)	
	Unemployed, peasant, retired, other		104 (55.0)	242 (55.0)	
**Surgical approach** **, n (%)**					*<.001* ^g^
	Video-assisted thoracoscopic surgery		176 (93.1)	358 (81.4)	
	Thoracotomy		13 (6.9)	82 (18.6)	
**Hospital type** **, n (%)**					.89^g^
	Provincial level		164 (86.8)	380 (86.4)	
	Municipal or county level		25 (13.2)	60 (13.6)	
**BMI (kg/m^2^)** **, n (%)**					.15^g^
	≤23.9		130 (68.8)	276 (62.7)	
	>23.9		59 (31.2)	168 (38.2)	
No smoking history^h^, n (%)			151 (79.9)	262 (59.5)	*<.001* ^g^
**Charlson Comorbidity Index** **score** **, n (%)**					*<.001* ^g^
	≤1		143 (75.7)	267 (60.7)	
	>1		46 (24.3)	173 (39.3)	
**Chest tube** **, n (%)**					*<.001* ^g^
	1		96 (50.8)	315 (71.6)	
	2		93 (49.2)	125 (28.4)	
**Disease type** **, n (%)**					*<.001* ^g^
	Nonlung cancer		16 (8.5)	82 (18.6)	
	Lung cancer with pTNM^i^ stage ≤I		145 (76.7)	217 (49.3)	
	Lung cancer with pTNM stage >I		28 (14.8)	141 (32.0)	

^a^P&P: paper and pencil.

^b^ePRO: electronic PRO (patient-reported outcome).

^c^No data missing for demographic and clinical characteristic variables.

^d^Statistically significant values are italicized (*P*<.05).

^e^Independent *t*-test.

^f^Wilcoxon 2-sample test.

^g^Chi-square test.

^h^Former or current smoker except no smoking history.

^i^pTNM: pathological tumor–node–metastasis.

### Compliance With Scheduled Assessments Over Time

**Figure 1:**
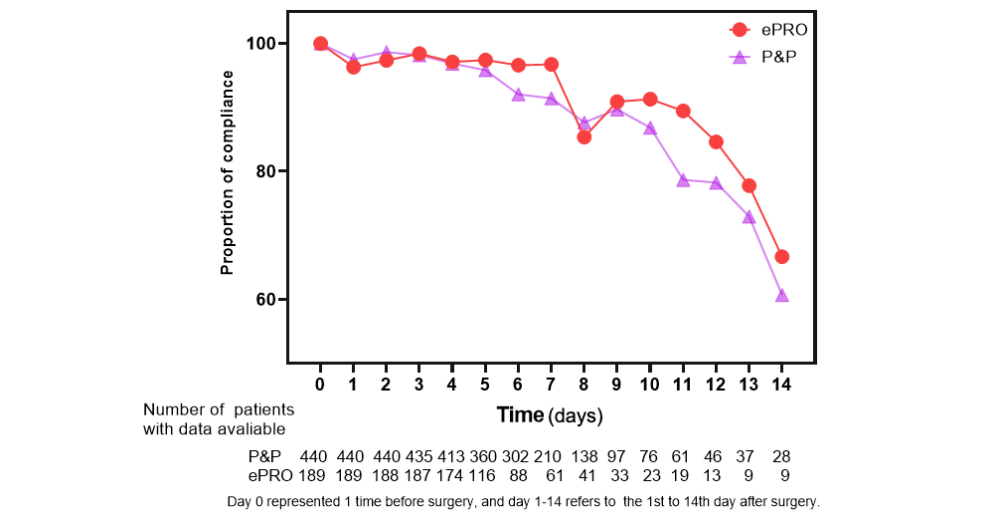
Proportion of patient compliance during 15 timepoints between different modes. ePRO: electronic PRO, P&P: paper and pencil, PRO: patient-reported outcome.

Of the 629 patients included in the analysis, 6.4% (28/440) of the patients in the P&P group and 3.7% (7/189) of the patients in the ePRO group withdrew from the studies during hospitalization. A total of 440 P&P patients generated 3347 PRO records, whereas 189 ePRO patients generated 1291 records. The compliance rates ranged from 67% (6/9 in POD 14) to 100% (189/189 before surgery) for the ePRO group and from 61% (17/28 in POD 14) to 100% (440/440 before surgery) for the P&P group over time (Figure 1).

### Error Patterns

We found that 49.4% (311/629) of the patients had at least one error, and a total of 1654 errors were identified. In Multimedia Appendix 1, missing items (64.69%, 1070/1654) and modifications without signatures (27.99%, 463/1654) were the top 2 most frequently observed errors, followed by multiple selections for 1 item (3.02%, 50/1654), missing patient signatures (2.54%, 42/1654), missing researcher signatures (1.45%, 24/1654), and missing completion dates (0.30%, 5/1654).

Multiple selections for a single item, modifications without patient or researcher signatures, and missing completion dates were only identified on P&P assessments, accounting for 32.77% (542/1654). Shown in Table 2, significant differences in the number of involved patients were found for the overall errors (ePRO: 30.2% [57/189] vs. P&P: 57.7% [254/440]; *P*<.001) and missing items (ePRO: 28.6% [54/189] vs. P&P: 55.0% [242/440]; *P*<.001). Very few “missing patient signature” errors were identified, and the proportion did not differ between the ePRO and P&P groups (2.1% [4/189] vs. 1.8% [8/440]; *P=*.76).

The error rates of each item (including missing items, modifications without signatures, and multiple selections for 1 item) within PRO instruments are presented in Multimedia Appendix 2. Overall errors and missing items were found in 4% of the items pertaining to distress and interferes (mood and relations) on both types of assessments (ePRO and P&P).

**Table 2:** Counts and proportion of involved patients and errors between assessment modes (ePRO^a^ vs. P&P^b^).

Error types	Errors (count), n		Involved patients, n (%)		*P* value^c^
	ePRO (n=189)	Paper (n=440)	ePRO (n=189)	Paper (n=440)	
Missing items	152	918	54 (28.6)	242 (55.0)	*<.001* ^d^
Modifications without signatures	0	463	0 (0)	140 (31.8)	
Multiple selection for 1 item	0	50	0 (0)	42 (9.5)	
Missing patient signatures	14	28	4 (2.1)	8 (1.8)	.76^e^
Missing researcher signatures	0	24	0 (0)	11 (2.5)	
Missing completion dates	0	5	0 (0)	3 (0.7)	
Overall errors	166	1488	57 (30.2)	254 (57.7)	*<.001* ^d^

^a^ePRO: electronic PRO (patient-reported outcome).

^b^P&P: paper and pencil.

^c^Statistically significant values are italicized (*P*<.05).

^d^Chi-square test.

^e^Fisher exact test.

### Factors Contributing to the Incidence of Errors

As shown in Table 3, patients with lower education levels (OR 1.82, 95% CI 1.22-2.72; *P*=.003), those treated at provincial hospitals (OR 4.73, 95% CI 2.18-10.25; *P*<.001), and those with severe disease (lung cancer with pTNM stage >I vs. nonlung cancer: OR 2.70, 95% CI 1.53-4.75; *P*<.001) were more likely to generate errors in the ePRO group. In the P&P group, a lower level of education (OR 1.39, 95% CI 1.20-1.62; *P*<.001), treatment in a provincial hospital (OR 3.34, 95% CI 2.10-5.33; *P*<.001), severe disease (lung cancer with pTNM stage >I vs. nonlung cancer: OR 1.63, 95% CI 1.33-1.99; *P*<.001), being younger (OR 1.47, 95% CI 1.15-1.88; *P*=.002), male sex (OR 1.41, 95% CI 1.12-1.78; *P*=.003), thoracotomy (OR 1.28, 95% CI 1.13-1.46; *P*<.001), a higher CCI score (OR 1.58, 95% CI 1.36-1.84; *P*<.001), and more chest tubes (OR 1.66, 95% CI 1.26-2.17; *P*<.001) were associated with a higher risk of errors. The details of risk factors for missing items in P&P and ePRO are shown in Multimedia Appendix 3.

**Table 3:** Factors associated with the error incidence rate of participants who filled out the (ePRO^a^ vs. P&P^b^) assessments^c^.

Factors		ePRO (n=189)		Paper-and-pencil mode (n=440)	
		OR^d^ (95% CI)	*P* value^e^	OR (95% CI)	*P* value^e^
Age (under 55 years vs. 55 years or older)		0.96 (0.48-1.93)	.91	1.47 (1.15-1.88)	*.002*
Gender (male vs. female)		0.93 (0.60-1.42)	.73	1.41 (1.12-1.78)	*.003*
Education (middle school or below vs. higher than middle school)		1.82 (1.22-2.72)	*.003*	1.39 (1.20-1.62)	*<.001*
Employment status (others vs. employed)		0.93 (0.65-1.34)	.71	1.15 (1.02-1.31)	.03
Surgical approach (thoracotomy vs. video-assisted thoracoscopic surgery)		1.95 (1.17-3.25)	.01	1.28 (1.13-1.46)	*<.001*
Hospital type (provincial level vs. municipal or county level)		4.73 (2.18-10.25)	*<.001*	3.34 (2.10-5.33)	*<.001*
BMI (>23.9 kg/m^2^ vs. ≤23.9 kg/m^2^)		1.36 (0.87-2.12)	.18	0.93 (0.79-1.10)	.40
Smoking status^f^ (yes vs. no)		0.70 (0.47-1.03)	.07	1.14 (0.90-1.46)	.28
Charlson Comorbidity Index score (>1 vs. ≤1)		2.40 (1.11-5.20)	.03	1.58 (1.36-1.84)	*<.001*
Chest tube (2 vs. 1)		0.57 (0.37-0.89)	.01	1.66 (1.26-2.17)	*<.001*
**Disease type**					
	Lung cancer with pTNM^g^ stage ≤I vs. nonlung cancer	1.21 (0.85-1.72)	.29	1.17 (0.88-1.57)	.28
	Lung cancer with pTNM stage >I vs. nonlung cancer	2.70 (1.53-4.75)	*<.001*	1.63 (1.33-1.99)	*<.001*
Postoperative hospital stay (6 days or above vs. under 6 days)		0.90 (0.56-1.47)	.69	1.12 (0.95-1.32)	.18

^a^ePRO: electronic PRO (patient-reported outcome).

^b^P&P: paper and pencil.

^c^Administration: generalized estimated equation model; α′=α/12=0.0042.

^d^OR: odds ratio.

^e^Statistically significant values are italicized (*P<*.05).

^f^Former or current smoker except no smoking.

^g^pTNM: pathological tumor–node–metastasis.

### Trajectories of Errors

**Figure 2:**
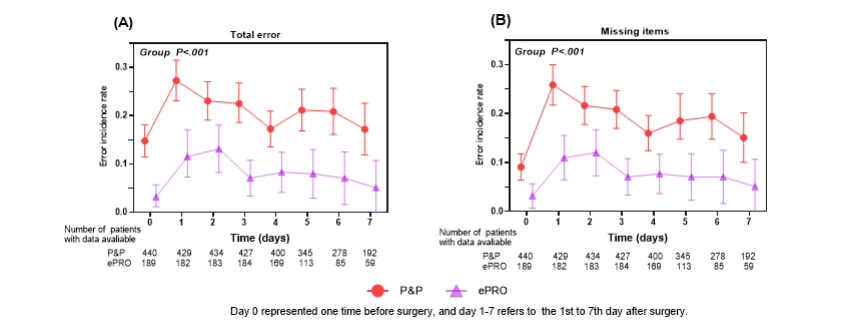
Error incidence rate of responded records with overall errors or item missing during 8 timepoints in hospital. ePRO: electronic PRO, P&P: paper and pencil, PRO: patient-reported outcome.

The trajectories of overall errors and missing items over time are illustrated for the ePRO and P&P assessments separately (Figure 2). In the P&P group, 14.8% (65/440) of patients made errors before surgery and then peaked on postoperative day 1 (POD 1; 117/440, 26.6%). The trajectory gradually decreased after surgery, but remained higher than that before surgery (17.2% [33/192] on POD 7). In the ePRO group, overall error was 3.2% (6/189) before surgery, followed by a continuous increase after surgery, peaking on POD 2 (13.1% [24/183]), and then gradually decreased but remained higher than that before surgery (POD 7 in 5.1% [3/59]). Similarly, missing items peaked on POD 1 in the P&P group (25.9% [111/429]) and on POD 2 in the ePRO group (12.0% [22/183]; Figure 2B). The details are presented in Table 4.

**Table 4:** Error incidence records of responded records with overall errors or item missing during 8 time points in hospital.^a^

Time (days)	Overall errors			Item missing			Completed patients	
	ePRO^b^ (n=189), n (%)	P&P^c^ (n=440), n (%)	*P* value^d,e^	ePRO^b^ (n=189), n (%)	P&P^c^ (n=440), n (%)	*P* value^d,e^	ePRO, n	P&P, n
0^f^	6/189 (3.2)	65/440 (14.8)	Mode=*.005*; time=*.03*; MT^g^=.69	6/189 (3.2)	40/440 (9.1)	Mode=*.005;* time=.06; MT^g^=.88	189	440
1	21/182 (11.5)	117/429 (27.3)		20/182 (11.0)	111/429 (25.9)		182	429
2	24/183 (13.1)	100/434 (23.0)		22/183 (12.0)	94/434 (21.7)		183	434
3	13/184 (7.1)	96/427 (22.5)		13/184 (7.1)	89/427 (20.8)		184	427
4	14/169 (8.3)	69/400 (17.3)		13/169 (7.7)	64/400 (16.0)		169	400
5	9/113 (8.0)	73/345 (21.2)		8/113 (7.1)	64/345 (18.6)		113	345
6	6/85 (7.1)	58/278 (20.9)		6/85 (7.1)	54/278 (19.4)		85	278
7	3/59 (5.1)	33/192 (17.2)		3/59 (5.1)	29/192 (15.1)		59	192

^a^Administration: generalized estimated equation (GEE) model.

^b^ePRO: electronic PRO (patient-reported outcome) (web-based).

^c^P&P: paper-and-pencil.

^d^Adjusted GEE model *P* values reported for time effect (as continual variable), mode effect (reference as P&P mode), interaction between mode and time effect (MT). All others are baseline covariant.

^e^Statistically significant values are italicized (*P*<.05).

^f^Day 0 represented the 1 time before surgery, and 1-7 refers to the 1st to day 7th after surgery.

^g^MT: interaction between mode effect and time effect.

The inflection time points were POD 2 for the ePRO assessment and POD 1 for the P&P assessment (Table 5). The incidence of errors on the ePRO assessments significantly increased from before surgery to POD 2 (estimate=0.51; *P*=.01, in model 2) and significantly decreased after POD 2 (estimate=–0.21; *P*<.001). However, errors on the P&P assessment significantly increased over the first 2 assessment time points (estimate=0.73; *P*<.001, in model 2) and slightly decreased after POD 1 (estimate=–0.10; *P*<.001). The details of item missing using 2-piecewise model are described in Multimedia Appendix 4.

**Table 5:** Two-piecewise regression analysis for each mode with overall errors during 8 time points in hospital.^a^

Mode		Overall errors			
		Estimate 1^b^ (standard error)	*P* value^c^	Estimate 2^d^ (standard error)	*P* value^c^
**Electronic** **PRO mode**					
	Model 1^e^	0.50 (0.20)	*.01*	–0.20 (0.04)	*<.001*
	Model 2^f^	0.51 (0.20)	*.01*	–0.21 (0.05)	*<.001*
	Model 3^g^	0.55 (0.19)	*.004*	–0.24 (0.05)	*<.001*
**Paper and pencil mode**					
	Model 4^e^	0.67 (0.06)	*<.001*	–0.08 (0.02)	*<.001*
	Model 5^h^	0.73 (0.05)	*<.001*	–0.10 (0.02)	*<.001*
	Model 6^g^	0.74 (0.05)	*<.001*	–0.11 (0.02)	*<.001*

^a^Administration: 2-piecewise model; inflection point, POD (postoperative day) 1 for P&P (paper and pencil) and POD 2 for ePRO (electronic PRO [patient-reported outcome]) .

^b^Estimate 1: piecewise regression coefficient on the left side of the inflection point, from before surgery to POD 2 in the ePRO mode or from before surgery to POD 1 in the P&P mode after surgery

^c^Statistically significant values are italicized (*P*<.05).

^d^Estimate 2: piecewise regression coefficient on the right side of the inflection point.

^e^Models 1 and 4: no adjustment.

^f^Model 2: adjustment for education, hospital level, and disease type.

^g^Models 3 and 6: adjustment for age group, gender, education, employment, surgical approach, hospital type, BMI, smoking history, Charlson Comorbidity Index score, chest tube, disease type, and postoperative hospital stay (days).

^h^Model 5: adjustment for age group, gender, education, surgical approach, hospital type, Charlson Comorbidity Index score, chest tube, and disease type.

## Discussion

### Principal Findings

For the first time, using data from studies that included PROs as major outcomes in the setting of thoracic surgery, we profiled 6 types and 2 trajectories of errors for PRO data collected daily using 2 major assessments (ePRO or P&P). Nearly one-fifth of the records and half of the patients had errors when longitudinal PROs were used as outcomes, even when a quality check was implemented immediately after the completion of data collection. We demonstrated that, compared with the P&P assessment, the ePRO assessment had higher compliance, which is necessary to maintain data quality, but needed more time for patient adaptation. In addition, significant selection bias was identified for the ePRO assessment, with younger, better educated, and more physically active patients being more likely to use. This quantification of the quality of frequently collected PRO data might support study design, data quality control, and data audits for surgical studies using PROs as outcomes and will help guide resource allocation when implementing PRO-based surgical patient care.

### Magnitude of Data Errors

The ePRO assessment had fewer errors. Over one-third of the errors occurred on P&P assessments, and these were errors that could be avoided by using the ePRO assessment. One study described missing items on anxiety questionnaires at 3 assessment points, and the results were as follows: 31.8% for P&P versus 2.08% for ePRO in the hospital [15]. Another study that investigated food-frequency questionnaires at 2 time points over 10 years revealed that the average rate of missing items on the form was 9% for P&P assessments and 3% for the electronic version [17]. The lower rates of errors observed in those 2 studies may be attributed to the lower frequency of measurement and the younger participants. Zeleke et al [42] analyzed 2492 records in an RCT involving healthy people and reported that 41.89% of the paper records and 30.89% of the electronic records had 1 or more types of data quality issues. Compared with those studies, our analysis, which had clear definitions of data inaccuracy and incompleteness, suggested the need for careful data quality monitoring plans in studies that require frequent assessments of PROs.

Missing items on assessments of PROs is a core issue and is nearly ubiquitous in clinical research. In this study, missing items accounted for a significant proportion (over three-fifths) of all errors. There is strong evidence that much of these missed items occur at random and are therefore almost impossible to eliminate in the real world [19,43,44]. Our results showed that missing items decreased by one-fifth when ePRO assessment was used, indicating that using this format could improve PRO data quality in further studies.

### Adaptation

The trajectories in errors significantly changed each day during the perioperative period, and different trends were observed for each assessment mode. Interestingly, constant trends, with an initial increase followed by a decrease over time, were observed with both the P&P and ePRO assessments in this study, whereas the results in a similar study showed random peaks and irregular trends when the data were presented according to the date of collection [42]. There are 2 possible reasons for the difference. First, the sequence of time points that this study followed merely ordered the data according to the natural progression of days, from day 1 of the survey to day 25 of the survey, whereas our analysis considered the sequence of response time points for each patient. Second, that study was performed at public health and demographic surveillance sites, whereas we targeted surgical patients in hospitals. By contrast, a learning curve usually occurs for the use of a new technological progress (reflected by a decreasing error rate) as a function of the accumulation of experience over time [45]. Errors peaked on POD 1 for P&P assessments and on POD 2 for ePRO assessments, suggesting that patients took less time to adapt to the former. Studies have reported that more experience and time are needed to adapt to electronic methods [46]. Basch et al [4] found that patients with prior computer use experience benefited relatively more from the web-based PRO monitoring and alerting system.

In general, paper-based assessments are expected to be the first choice [17]. P&P is still a major method of assessment in clinical research, especially for older, poorer, or sicker patients. To accommodate a more representative patient set, ePRO needs to be made more user-friendly. For example, reducing the complexity of operating the interface, adding or optimizing automated interactive voice functions, and designing automated telephone systems outside of the hospital should be considered [47]. Given the convincing equality in measuring patient perception, a mixed-mode system involving both P&P and ePRO assessments could be a better choice. The preferred option might be ePRO assessments, with P&P assessments as the secondary choice for almost all patients in clinical studies.

### What Are the Factors That Influenced Data Quality?

In this analysis, patients treated in provincial hospitals were more likely to produce poor-quality PRO data regardless of whether they used the P&P or ePRO assessments. The explanation was that the majority of patients and heaviest clinical workload are concentrated in provincial hospitals in China [48]. Medical staff in provincial hospitals are busier than those in municipal or county-level hospitals in routine clinical practice, which may result in less effort given to data monitoring. For any patient-centered practice or research, more efforts are required to obtain better data availability and accuracy in health care system. Other shared factors affecting errors in both modes are education level and physical status. Therefore, we suggest that there should be prespecified means of assistance provided to participants who are more likely to struggle to complete the assessments [49]. For example, measures might be taken to help patients complete scheduled PRO assessments when they have greater difficulties filling in the form [50]. Compared with P&P assessments, ePRO assessments had fewer risk factors for poor data quality. One possible explanation might be the homogeneous population using ePRO assessments due to biased sampling, as their use requires a certain level of education [51].

### Limitations

We acknowledge that the results are limited by the potential sample bias and the differences in study designs and data collection tools. We may have overestimated the differences between the P&P and ePRO assessments because RCTs are managed better than observational studies [52], although the same team of clinical coordinators and same data quality control standard operating procedure were used for both projects. A second limitation is that the data were only collected during hospitalization because almost all ePRO assessments were administered after discharge in our study. This is similar to a study that showed that the ePRO assessment was more cost-effective and user friendly for clinical staff and patients [16,31] and suggested that there is a trend in the implementation of ePRO assessments in clinical research. Finally, this study lacks evidence of the equivalence of the data collected with the 2 forms of assessment, and therefore cannot state whether the data collected with the 2 assessments are equally valid. Further research is needed to confirm these results.

### Conclusions

In conclusion, this study with substantial sample and longitudinal design demonstrates the pros and cons of the 2 most commonly used methods (ePRO and P&P), which will help promote web-based patient care [53]. It is possible to improve the quality of longitudinal PRO data by using web-based assessments. Although ePRO was found to be superior to P&P in terms of data quality, ePRO-related sampling bias should be taken into consideration when designing clinical research using longitudinal PROs as a major outcome.

Alternatively, providing the option of using either the ePRO or the P&P assessment would improve the representativeness of samples if the comparativeness of the data obtained with the ePRO and P&P assessments is confirmed by well-designed equivalence studies.

## Appendix

Multimedia Appendix 1 Description of the error pattern.

Multimedia Appendix 2 Item missing and overall errors within each item of MD Anderson Symptom Inventory Lung Cancer Module (MDASI-LC) and quality of life (QOL).

Multimedia Appendix 3 Factors associated with the item missing incidence rate, of participants who filled out the ePRO (ePRO: electronic PRO [patient-reported outcome]), P&P (paper and pencil) and overall modes.

Multimedia Appendix 4 Two-piecewise regression analysis for each mode with item missing during 8 days in hospital.

## Abbreviations

CCI: Charlson Comorbidity Index
ePRO: electronic PRO
GEE: generalized estimating equation
MDASI-LC: MD Anderson Symptom Inventory Lung Cancer Module
OR: odds ratio
P&P: paper and pencil
POD: postoperative day
PRO: patient-reported outcome
pTNM: pathological tumor–node–metastasis
QIC: quasi-likelihood under the independence model criterion
QOL: quality of life
RCT: randomized controlled trial
VATS: video-assisted thoracoscopic surgery
