# Supplementary figures and images for: Data Quality of Longitudinally Collected Patient-Reported Outcomes After Thoracic Surgery: Comparison of Paper- and Web-Based Assessments
Source: J Med Internet Res. 2021 Nov 9;23(11):e28915. doi: 10.2196/28915 (PMC8663677; doi:10.2196/28915)

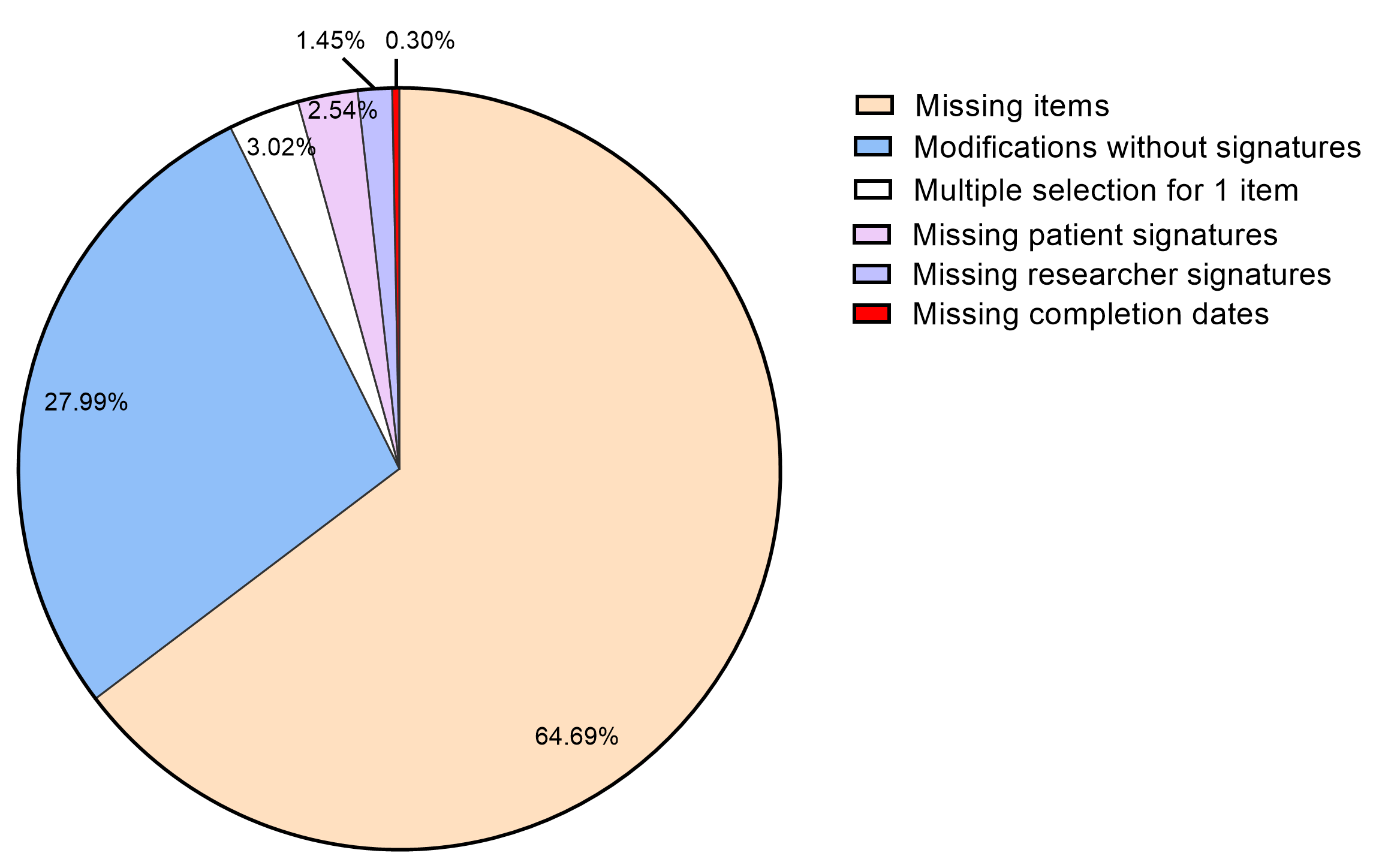

Supplement: Multimedia Appendix 1 [file jmir_v23i11e28915_app1.png]
