# Supplementary material for: Data Quality of Longitudinally Collected Patient-Reported Outcomes After Thoracic Surgery: Comparison of Paper- and Web-Based Assessments
Source: J Med Internet Res. 2021 Nov 9;23(11):e28915. doi: 10.2196/28915 (PMC8663677; doi:10.2196/28915)
Supplement: Multimedia Appendix 2 [file jmir_v23i11e28915_app2.doc]

**Table S1. Item missing and overall errors within each item of MDASI-LC^a^ and QOL^b^.**

| Items | | Item missing, n(%)c | | Overall errorsd, n(%)c | | | |
| --- | --- | --- | --- | --- | --- | --- | --- |
|  |  | ePRO^d^ | P&P^e^ | ePRO^d^ | | P&P^e^ |  |
| **MDASI-LC Symptoms PART I. CORE Items** | | | | | | | |
|  | Pain | 2(1.3) | 29(3.2) | 2(1.3) | 52(3.6) | | |
|  | Ftigue(tiredness) | 4(2.6) | 24(2.6) | 4(2.6) | 43(3.0) | | |
|  | Nausea | 4(2.6) | 52(5.7) | 4(2.6) | 90(6.3) | | |
|  | Disturbedsleep | 4(2.6) | 31(3.4) | 4(2.6) | 53(3.7) | | |
|  | Distressed(upset) | 7(4.6) | 36(3.9) | 7(4.6) | 61(4.3) | | |
|  | Shortnessofbreath | 4(2.6) | 26(2.8) | 4(2.6) | 46(3.2) | | |
|  | Problemofrememberingthings | 10(6.6) | 25(2.7) | 10(6.6) | 44(3.1) | | |
|  | Lackofappetite | 1(0.7) | 31(3.4) | 1(0.7) | 58(4.1) | | |
|  | Drowsy(sleepy) | 4(2.6) | 21(2.3) | 4(2.6) | 33(2.3) | | |
|  | Drymouth | 5(3.3) | 27(2.9) | 5(3.3) | 46(3.2) | | |
|  | Sad | 7(4.6) | 29(3.2) | 7(4.6) | 48(3.4) | | |
|  | Vomiting | 7(4.6) | 20(2.2) | 7(4.6) | 36(2.5) | | |
|  | Numbnessortingling | 7(4.6) | 12(1.3) | 7(4.6) | 22(1.5) | | |
| **MDASI-LC Symptoms PART I. Lung Cancer-Specific Items** | | | | | | | |
|  | Coughing | 5(3.3) | 27(2.9) | 5(3.3) | 40(2.8) | | |
|  | Constipation | 7(4.6) | 26(2.8) | 7(4.6) | 41(2.9) | | |
|  | Sore throat | 3(2.0) | 17(1.9) | 3(2.0) | 27(1.9) | | |
| **MDASI-LC Symptoms PART II. Interfered Items** | | | | | |  |  |
|  | Activity | 2(1.3) | 38(4.1) | 2(1.3) | 68(4.8) | | |
|  | Mood | 7(4.6) | 47(5.1) | 7(4.6) | 76(5.3) | | |
|  | Work | 2(1.3) | 63(6.9) | 2(1.3) | 99(6.9) | | |
|  | Relations with other people | 7(4.6) | 41(4.5) | 7(4.6) | 66(4.6) | | |
|  | Walking | 5(3.3) | 39(4.3) | 5(3.3) | 61(4.3) | | |
|  | Enjoyment of life | 3(2.0) | 24(2.6) | 3(2.0) | 36(2.5) | | |
| **Single-item QOL** | | 45(29.6) | 233(25.4) | 45(29.6) | 285(19.9) | | |
| **Total** | | 152 | 918 | 168 | 1488 | | |

^a^MDASI-LC,: MD Anderson Symptom Inventory of Lung Cancer, includes (1): PART I. CORE Items; (2): PART I. Lung Cancer-Specific Items; (3): PART II. Interfered Items.

^B^QOL: single item quality of life.

^c^Data are presented on number and number of total count (n[%]).

^d^Overall error includes item missing, modification without signature and multiple selection.

^e^ePRO: electronic PRO.

^f^P&P: paper and pencil.
