# Supplementary material for: Data Quality of Longitudinally Collected Patient-Reported Outcomes After Thoracic Surgery: Comparison of Paper- and Web-Based Assessments
Source: J Med Internet Res. 2021 Nov 9;23(11):e28915. doi: 10.2196/28915 (PMC8663677; doi:10.2196/28915)
Supplement: Multimedia Appendix 3 [file jmir_v23i11e28915_app3.doc]

**Table S2.**  Factors associated with the item missing incidence rate, of participants who filled out the ePRO^a^, P&P^b^ and overall modes^c^.

| Factors | | ePRO^a^ (n=189) | | P&P^b^ (n=440) | |
| --- | --- | --- | --- | --- | --- |
|  |  | OR^d^ (95% CI) | *P*value | OR^d^ (95% CI) | *P* value |
| Age (under 55 years vs. 55 years or older) | | 0.86(0.39-1.91) | .71 | 1.48 (1.13-1.94) | .005 |
| Gender (male vs. female) | | 1.03 (0.56-1.89) | .92 | 1.53 (1.21-1.94) | *<.001* |
| Education (middle-school graduate or below vs. over middle-school graduate) | | 2.01 (1.39-2.92) | *<.001* | 1.43 (1.17-1.72) | *<.001* |
| Employment status (others vs. employed) | | 0.89 (0.57-1.39) | .60 | 1.12 (0.95-1.30) | .17 |
| Surgical approach (thoracotomy vs. video-assisted thoracoscopic surgery) | | 2.13 (1.24-3.66) | .01 | 1.24 (1.06-1.46) | .01 |
| Hospital type (provincial level vs. municipal or county level) | | 4.14 (2.14-8.01) | *<.001* | 7.11 (3.83-13.18) | *<.001* |
| BMI (>23.9 kg/m^2^ vs. ≤23.9 kg/m^2^) | | 1.47 (0.92-2.34) | .10 | 0.95 (0.79-1.14) | .57 |
| Smoking status^e^ (yes vs. no) | | 0.77 (0.53-1.13) | .19 | 1.23 (0.95-1.59) | .12 |
| Charlson Comorbidity Index score (>1 vs. ≤1) | | 2.61 (1.17-5.82) | .02 | 1.48 (1.29-1.70) | *<.001* |
| Chest tube (2 vs. 1) | | 0.70 (0.44-1.10) | .12 | 1.84 (1.42-2.37) | *<.001* |
| **Disease type** | |  |  |  |  |
|  | Lung cancer with pTNM^f^ stage ≤I vs. nonlung cancer | 1.48(0.71-3.09) | .30 | 1.12 (0.83-1.51) | .46 |
|  | Lung cancer with pTNM stage >I vs. nonlung cancer | 3.77(1.62-8.77) | *.002* | 1.60 (1.33-1.93) | *<.001* |
| Postoperative hospital stay (6 days or above vs. under 6 days) | | 0.77 (0.52-1.14) | .19 | 1.13 (0.94-1.37) | .20 |

Statistically significant values are given in italicize (*P*＜.0034).

^a^P&P: paper and pencil.

^b^ePRO: electronic PRO.

^c^Administration: generalized estimated equation model; α′=α/12=0.0042.

^d^OR: odds ratio.

^e^Former or current smoker except no smoking.

^f^pTNM: pathological tumor–node–metastasis.
