# Supplementary material for: Data Quality of Longitudinally Collected Patient-Reported Outcomes After Thoracic Surgery: Comparison of Paper- and Web-Based Assessments
Source: J Med Internet Res. 2021 Nov 9;23(11):e28915. doi: 10.2196/28915 (PMC8663677; doi:10.2196/28915)
Supplement: Multimedia Appendix 4 [file jmir_v23i11e28915_app4.doc]

**Table S3.** Two-piecewise regression analysis for each mode with item missing during 8 days in hospital^a^.

| Mode | | Item missing | | | |
| --- | --- | --- | --- | --- | --- |
|  |  | Estimate1^b^ (standard error.) | *P* value | Estimate2^c^ (standard error.) | *P* value |
| **Electronic PRO mode** | | | | | |
|  | Model1^d^ | 0.47 (0.19) | *.011* | -0.19 (0.04) | *<.001* |
|  | Model2^e^ | 0.49 (0.19) | *.011* | -0.21 (0.05) | *<.001* |
|  | Model3^f^ | 0.52 (0.19) | *.004* | -0.22 (0.05) | *<.001* |
| **Paper and pencil mode** | | | | | |
|  | Model4^d^ | 1.16 (0.06) | *<.001* | -0.93 (0.02) | *<.001* |
|  | Model5^g^ | 1.24 (0.06) | *<.001* | -0.11 (0.02) | *<.001* |
|  | Model6^f^ | 1.25 (0.06) | *<.001* | -0.12 (0.03) | *<.001* |

Statistically significant values are given in italicize (P＜0.05).

^a^Administration: 2-piecewise Model; inflection point, POD 1 for P&P and POD 2 for ePRO.

^b^Estimate 1: piecewise regression coefficient on the left side of the inflection point, from before surgery to POD 2 in the ePRO mode or from before surgery to POD 1 in the P&P mode of post surgery

^c^Estimate2: piecewise regression coefficient on the right side of the inflection point.

^d^Models 1 and 4: no adjustment.

^e^Model 2: adjustment for education, hospital level, and disease type.

^f^Models 3 and 6: adjustment for age group, gender, education, employment, surgical approach, hospital type, BMI, smoking history, Charlson Comorbidity Index score, chest tube, disease type, and postoperative hospital stay (days).

^g^Model 5: adjustment for age group, gender, education, surgical approach, hospital type, Charlson Comorbidity Index score, chest tube, and disease type.
